# Spider webs inspiring soft robotics

**DOI:** 10.1098/rsif.2020.0569

**Published:** 2020-11-11

**Authors:** Fritz Vollrath, Thiemo Krink

**Affiliations:** 1 Department of Zoology, University of Oxford, Mansfield Road, Oxford OX1 3ZS, UK; 2 Department of Computer Science, Aarhus Universitet, Åbogade 34, 8200 Aarhus, Denmark

**Keywords:** spider web, behaviour, stigmergy, rule systems, evolutionary algorithms, light-weight construction

## Abstract

In soft robotics, bio-inspiration ranges from hard- to software. Orb web spiders provide excellent examples for both. Adapted sensors on their legs may use morphological computing to fine-tune feedback loops that supervise the handling and accurate placement of silk threads. The spider's webs embody the decision rules of a complex behaviour that relies on navigation and piloting laid down in silk by behaviour charting inherited rules. Analytical studies of real spiders allow the modelling of path-finding construction rules optimized in evolutionary algorithms. We propose that deconstructing spiders and unravelling webs may lead to adaptable robots able to invent and construct complex novel structures using relatively simple rules of thumb.

## 1. Background

Soft robotics or bio-robotics is a novel rapidly growing field that combines biological insights with engineering solutions in order to address fundamental questions of real-life demands [1–3]. Solutions in conventional robotics rely on principal design criteria of repeatability, precision, accuracy and strength. Nature, by contrast, uses combinations of fundamentally soft materials to construct highly flexible bodies with rule-of-thumb minds well adapted to tasks of making-do. The octopus robot is a perfect example of the issues involved in constructing a bioinspired soft-robot, both intellectually and practically [4–6]. Integrating the body's hardware (especially if soft and nonlinear) and the controller's software can present high-level challenges [7]. In this context, the spider and its web present an interesting paradigm because they act as a unit with the animal's behaviour creating a structure that, as an extension of the spider's body, takes over certain functions both sensory and ‘muscular’ [8]. This extension is relatively inexpensive to make, is self-tuning through its silks and, being renewed daily, readily matches changing conditions.

Web-building spiders provide interesting examples of model systems for the interface of Nature and engineering. The spider's orb web embodies the decision rules of a complex orientation behaviour that relies on navigation and piloting [8]. As an extended phenotype, the web is the frozen record of a long sequence of behaviour patterns [9,10] that follow inherited rules evolved over millions of years [11,12]. Analytical studies of real spiders followed by computer modelling allow us to unravel as well as test and deploy key path-finding and construction rules in a virtual environment [13,14]. Here we combine a virtual spider robot (controlled by a rule system) with an evolutionary algorithm (using cost–benefit evaluations). This allows us to explore the flexibility inherent in a natural system that relies heavily on emergent properties, which is a key feature of stigmergy [15]. Thus, the spider and its web provide innovative procedural insights for creating highly adaptable and efficient robots able to invent and construct complex novel structures using relatively simple rules of thumb.

**Figure 1.**
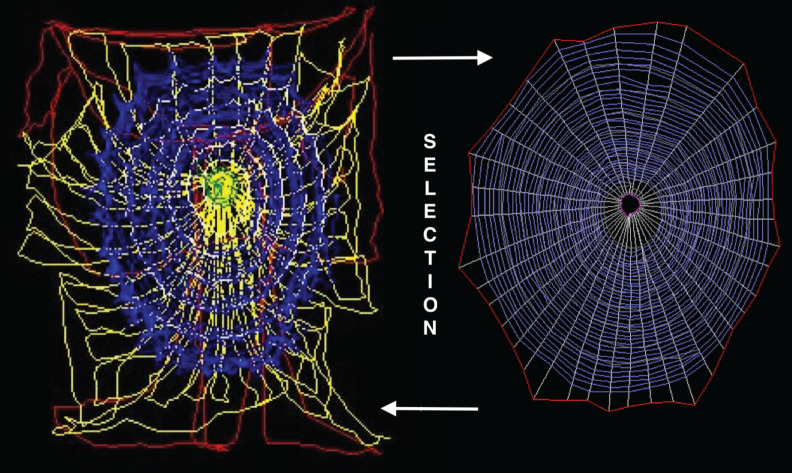
Behaviour and structure evolve together in the spider's web. The building path (on left) is a track of detours, while the final web structure (on right) represents the essence of the path frozen in a network of silk. Here, the construction actions and threads of the orb spider *A. diadematus* are colour coded. The behaviour (left) first anchors and puts up the frame threads (red) filled in by a tight wheel of spokes (yellow). These radials are then linked by a scaffolding auxiliary spiral (white) laid from the inside out. This geometric temporary spiral is superseded (and in the process cut down) by the arithmetic permanent and sticky capture spiral (blue) built from the outside in ending with the spider at the hub surrounded by its evenly meshed, virgin capture net. Thus, in the final web (right) the stiffening scaffolding spiral is absent (now radials coded in white). Selection acts on the web through (i) costs of the assembly (time, silk, risk of predation) and (ii) the benefits of the trap, i.e. prey caught, creating an evolutionary feedback loop between the two phenotypes of fleeting behaviour (left) and semi-permanent structure (right). For details, see [8,10].

In robotics terms, this natural enlargement of the spider's body is tangible structure (hardware) created by behaviour (software) manipulating silk (hardware) in an iterative process of growing complexity and emergent properties (figure 1). Some of that complexity is mitigated by the structure acting as a dynamic information filter considered to be morphological computing by roboticists [16–18]. Thus, both the web-making process and its outcome provide an interesting paradigm for roboticists because the combination of spider and web constitutes a complex and in parts complicated arrangement (or device) in which emergent properties become an integral part of the overall system. Webs are natural designs where diversity and quality can be studied in great detail [8,10] and can also be easily translated into the virtual reality of a digital cyber world [13,14,19–21].

## 2. Results

A web-building spider—such as the orb spider *Araneus diadematus* (commonly called the garden cross spider)—faces interesting challenges, and so does the bioinspired model spider such as, in our case, the cyber spider *Theseus*. In nearly all web spiders the fundamental source of input is haptic and relies on touch and vibrations. In addition, the animal also uses positional (kinaesthetic) information of each leg provided by internal (proprioceptive) sensory organs in combination with a memory stack on the path already travelled allowing idiothetic path integration [8]. In *A. diadematus*, like most other orb spiders that still have all their legs, the two pairs of front legs detect the presence of threads and measure distances while the spinneret lays and affixes the silk assisted by the fourth pair of legs with the third pair holding onto the supporting threads.

The virtual web of our cyber spider *Theseus* is a two-dimensional network of rigid ‘segments’ connected to others at ‘junctions’. *Theseus* crawls from one attachment point to the next with its web building being divided into a series of cycles each consisting of a crawl along existing threads (navigation) and an attachment or removal of a thread (construction). During each cycle, a new junction is added to connect a new segment to its predecessor. Local web geometry and global web position are factors for the placement of attachment points; this information the spider must sense itself. Local web geometry is found, for example, during capture spiral construction, by the position of the radials (leading to the hub) and auxiliary spiral as well as the distance to the previous turn of the capture spiral. Global web position is given by the direction of gravity (the vertical), although both real and simulated webs can be built in zero gravity with somewhat more symmetric features.

Here, we discuss two slightly different versions of *Theseus* dedicated to two different questions. Focusing on behaviour, we employ *Theseus AD* (the AD invoking *A. diadematus*) which allows us to study and test in detail the rules used by a real *Araneus* to position the capture spiral—including the use of shorter regenerated legs, which probes the importance of length measurements. To explore the effects of selection and adaptation on web construction, we employ *Theseus EVO*—with EVO standing for *evolution*. This allows us to explore the concept of encoding web-building rules into a genetic algorithm in order to examine the response to selection pressures on overall web parameters and structure. Both rule details and rule optimization will be of considerable importance in any attempt to build a truly bioinspired robot spider.

### 2.1. Adaptations in a web-building control system

**Figure 2.**
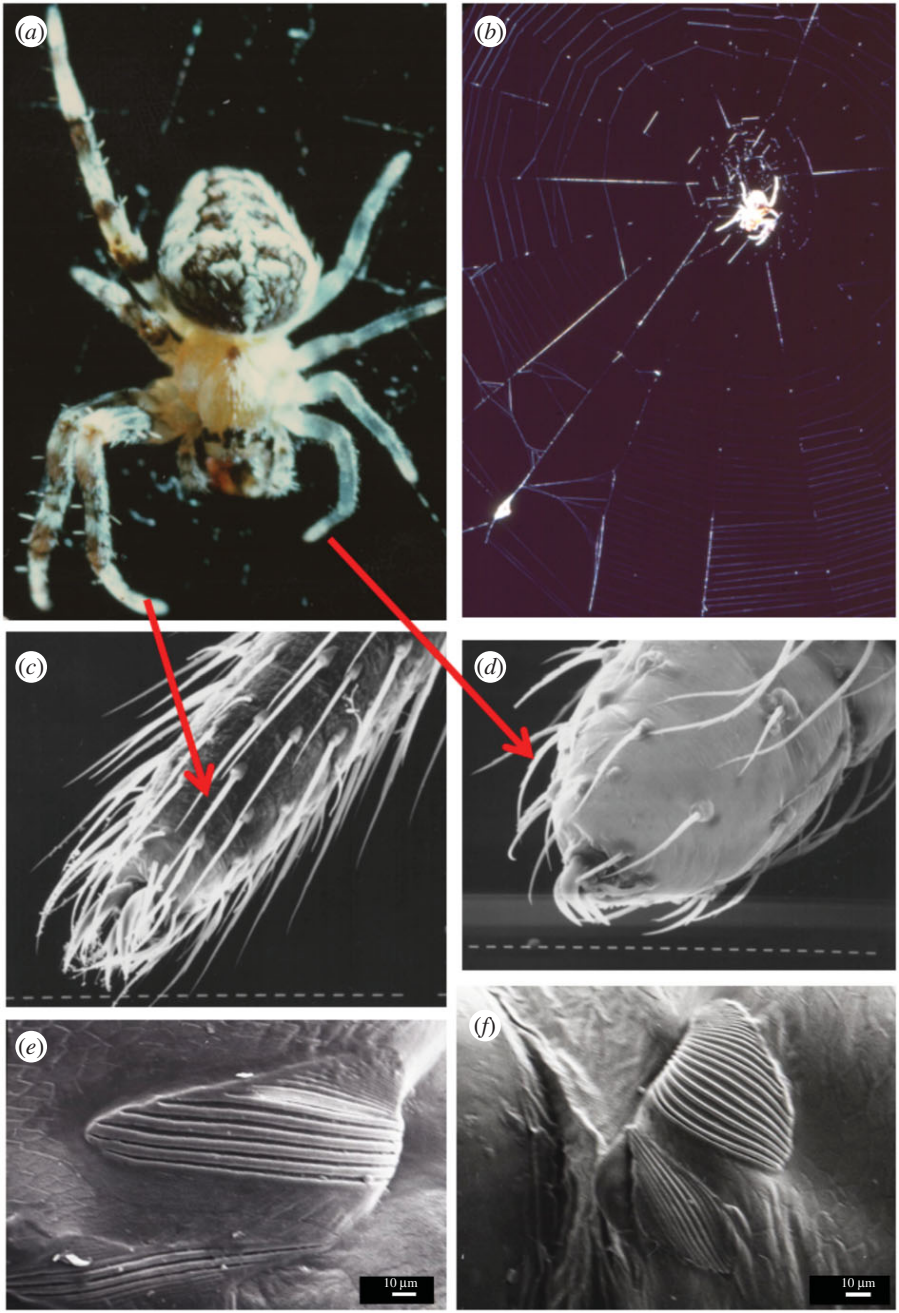
Regeneration of legs in *Araneus*. (*a*) Some spiders, like this common garden cross spider *A. diadematus*, regenerate lost limbs after voluntary autotomy and are able to build and also operate. (*b*) A perfect web already the very day of their moult when the legs are still untried [22]. These new legs are much stubbier and less hairy than the normal legs and with slightly smaller and modified claws (*c*,*d*). Importantly, in lyriform organs, the physical form determines the sensory function [25] and on the regenerated legs, they differ significantly in shape [24] with implications on sensing as well as feedback on leg positions (*e*,*f*). Note that (*a*) shows the dorsal and (*b*) the ventral view of the spider; also note the wrapped prey left *in situ* in the web, a natural behaviour. See figure 4 for a web built with regenerated legs.

Fortunately for investigations into the spider's behaviour algorithms, some web-building spiders are able to regenerate lost legs that are also fully functional on first use [22]. Not all web spiders do this but instead actively supress regeneration [23], which tells us something about the sensor and motor requirements and hand-shaking necessary to control and supervise the fine movements and handling associated with web building [24]. In *A. diadematus*, the regenerated legs are much shorter and stubbier than those they replace yet already fully functional hours after emerging from the precursor so far dormant under the hip stump of the old discarded leg [22]. Importantly, not only are these legs overall shaped differently from their un-regenerated counterparts but key sensory organs, the lyriforms, on these legs have different morphologies, too (figure 2).

**Figure 3.**
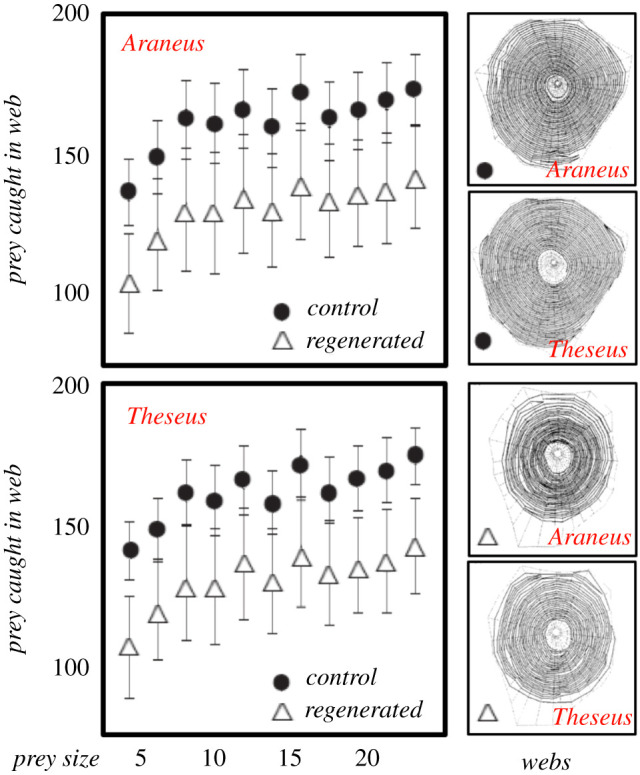
Comparing the webs of real spider *A. diadematus* and cyber spider *Theseus v2*. Both had either all legs normal lengths (control) and or had the two front legs on one side regenerated to roughly half size (regenerated). A sample web for each treatment is shown on the right. Both real and virtual spiders were able to construct functional webs with regenerated (and thus shortened) forelegs on one side of *Araneus* that affect the regularity of the capture spiral negatively; as they also did on *Theseus*. While regularity suffers, and a few other web parameters are also affected following leg regeneration, the overall function of the webs seems to be rather robust (for details, see [13]). This feature has implications for the validity of using the concept of leg regeneration (and the related concept of morphological computing) in the web-building algorithm of our spider model. For more details, see appendix A and [13].

The modifications in the lyraform organs are a bit of a give away because in these highly geometric organs the physical form maps directly onto the signal output [25], suggesting that the evolutionary tuning of anatomic details may involve morphological computing via a matched filter set-up [23,24]. In short, it is not unreasonable to assume that here we have an example of a highly adapted control system: the tuned sensor modifies the information, which allows the central processor (the animal's brain) to run its normal controller program to instruct the actuators. The ease-of-use robustness would suggest that the feedback matching of the incoming and outgoing signals is also done by the adapted sensors [23]. Whatever the mechanism, *Araneus* spiders with regenerated legs cannot only build highly functional webs (figure 3) but also operate them very well indeed [13].

**Figure 4.**
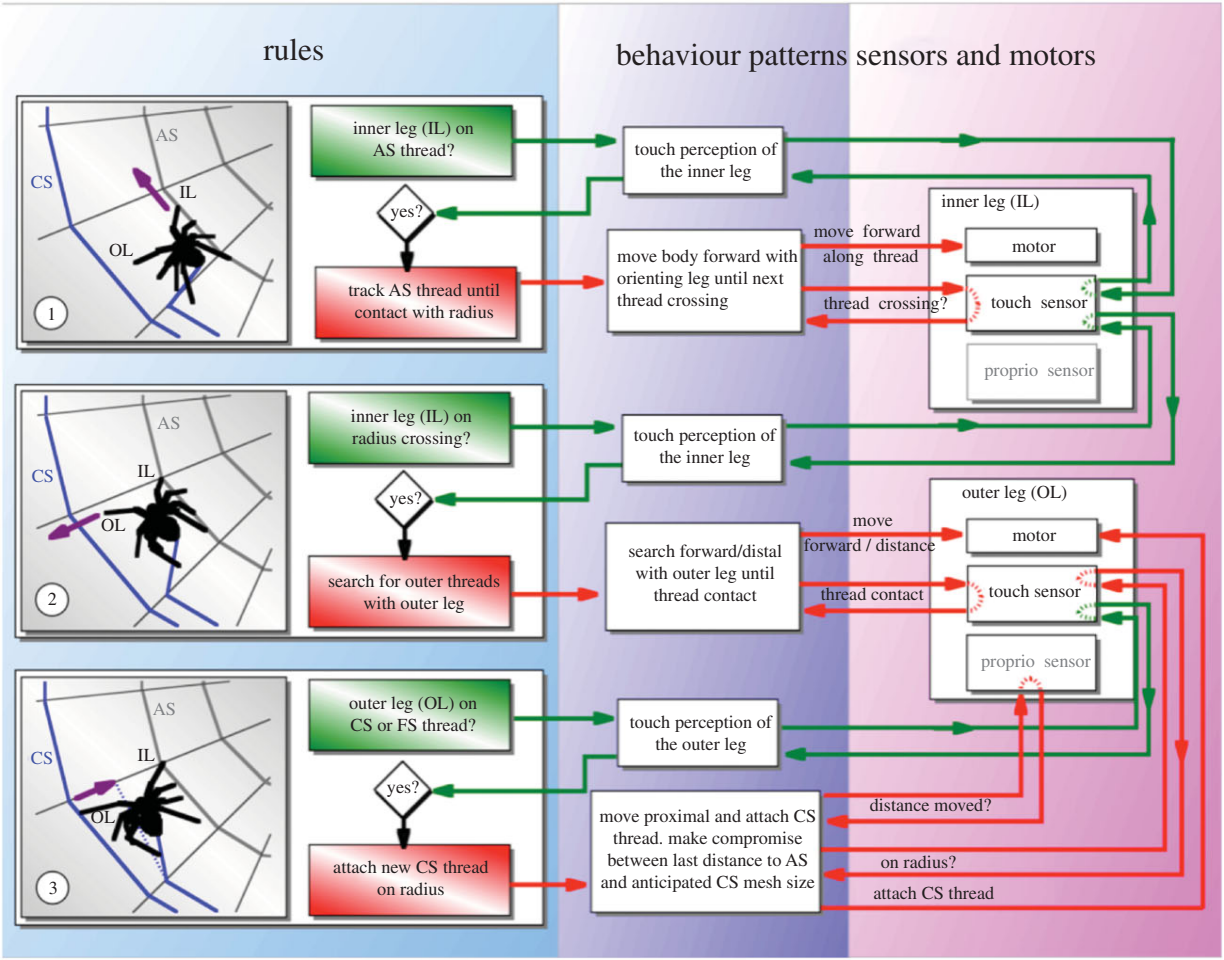
Analysing web spiral construction by an orb-weaving spider. The translation of the behaviour into hypothetical rules for decisions, sensors and actuators is possible because of detailed studies of capture spiral construction in an *A. diadematus* [8,21,26]. For details, see electronic supplementary material, appendix SB, that provides the outline of downloadable application.

Comparison of natural webs with virtual webs built by our cyber spider *Theseus* allows us to probe and test possible web algorithms and details of embedded decision rules on the placement of threads (figure 4). In both cases, the position of a fibre junction determines the position of the next one and thus in sequence govern the emergence of a fibre network and tension field, which in turn affects (and perhaps even determines) the overall geometry of the final web.

### 2.2. Spider web building encoded as a set of decision rules

In evolution, some traits change faster than others with some features being more variable and thus adaptable than others [27]. Behaviour is an outlier among most inherited traits, in that it shows exceptional flexibility in its expression [28,29]. Web spiders provide an outstanding example for this trait because here fleeting behaviour is frozen in time and recorded in structures that can be measured and probed in great detail [30]. In technological terms, the spider operates as an autonomous robot (without GPS and controller supervision) relying on an internal guidance system supported by feedback from a self-built environment with emergent properties. A simple set of rules or behaviour patterns invokes repeated local interactions with an environment that is both modified and used as feedback for local orientation (figure 4).

Clearly, the spider and its web provide an outstanding example to study a number of concepts of importance to robotics. On the one hand, there is the behaviour itself, i.e. the exact makeup of the decision rules that the animal uses to build its web as well as the evolution of the path-finding rules used to orient in the emerging structure. On the other hand, there is the question whether unravelling this kind of behaviour might be useful for insights into designing robust robots to operate in challenging and information-deprived environments. Here, we note that regarding spatial awareness, the real spider seems to be operating in a self-centred domain, i.e. piloting and navigating in a coordination system that is polar rather than Cartesian [8], which would be of interest for technology transfer into specific technical applications such as self-driving cars [31].

### 2.3. Analysis of *Araneus* web building

The garden cross spider *A. diadematus* readily adapts its orb web architecture to environmental conditions with many factors affecting web construction and consequently web structure [32,33]. Thus, although the building behaviour follows a fixed general schema, it clearly has ample scope for special local adaptations. As a process, web-building behaviour involves orientation (landmark piloting and vector navigation) in three-dimensional space combined with the sub-millimetre placement and manipulation of micro-sized silken threads. Rules of orientation guide the animal's path, while rules of manipulation determine points where threads are connected.

For unravelling the building algorithm, the regeneration of lost legs permits detailed analysis of particular legs as measuring devices. It emerges that, during web construction, *Araneus* uses its first pair of legs to measure certain distances, like the spacing of joints on a radial in the capture spiral. By contrast, geometric dimensions do change when angles (rather than distances) are measured by the shorter regenerated legs during handling and positioning of threads and joint [13,22].

Although in the finished web we see only the threads and joints, detailed studies of all the behaviour patterns involved in web building allow us to infer from the positioning of the joints not only the path but also the rules of placement [8,26] (figure 1). The process consists of a sequence of behaviour patterns that rely on measuring geometric parameters such as distances and angles, and include path integration in a spider internal guidance system using spatial information that is acquired both locally and globally [8].

Repeating experiments on different genotypes allowed us to dissect the action and expression of the hidden rules (the flow of information) that guide and control the behaviour, which creates the phenotype of the web. Detailed analysis of experimental perturbations of building spiders, ideally of sufficiently different genotypes, then allowed us to link rule action with parameters of the web-building algorithm and to test these hypothetical links in our computer model spider *Theseus*.

### 2.4. *Theseus* cyber spider web building and web evolution

**Figure 5.**
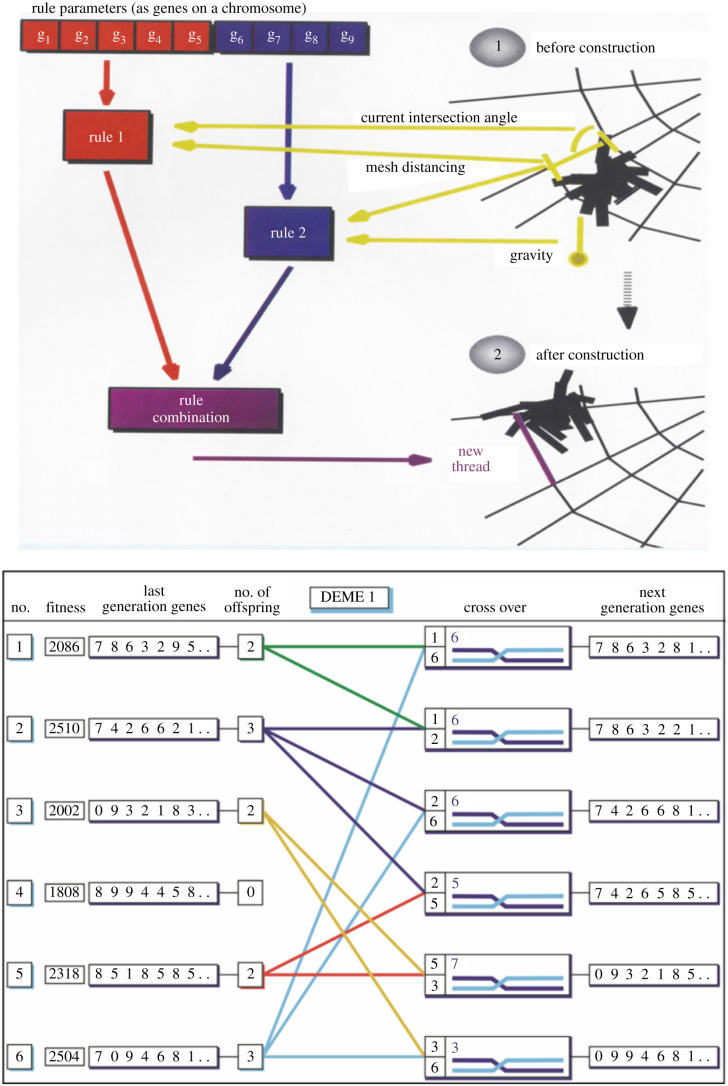
*Araneus* spiral construction encoded in hypothetical genes implemented in *Theseus.* Rule-based system (top) of *Thesus AD* for the construction of a node between the capture spiral and a radius based on studies of *Araneus* behaviour and thread geometry in the web. For the rule interactions, see figure 4. *Theseus* cyber genetics (bottom) has a double set of chromosomes that carry the rules represented as alleles. It deploys *mutation* to generate rule modifications and maintain variety as well as using *recombination* to let the two rule sets interact. *Selection* (number of offspring) discovers the top algorithms for a particular set of cost–benefit correlations. Mutation (settings not shown here) generates novel traits or re-discovers old (lost) traits with a high rate severely disrupting an established and functional gene network. Recombination is a highly efficient, and implicitly parallel, search that can bring together advantageous as well as disadvantageous gene states (alleles) with a high rate leading to a loss of traits. For more on the selection parameters, see figure 6.

*Theseus* is a virtual spider modelled on the common garden spider *A. diadematus*. *Theseus*' brain comprises a controller with rules and an interpreter [13,19–21,26]. Each rule requires a condition and responds with an action (figure 5). A web-building simulation consists of a sequence of rule-cycles whereby the interpreter tests the conditions for all rules, selects rules that satisfy the present conditions and performs the appropriate action(s). Importantly, small changes to the rule set allow the study of their effect on the web, such as the simulation of the famous drug effects [34] by tweaking the interaction between the decision parameters angle and distance to become unstable. Experiments conducted on *Araneus* web building were simulated with *Theseus* allowing us to test specific hypotheses and to probe into the way local and global cues could be used to explore the role of kinaesthetic and idiothetic factors.

**Figure 6.**
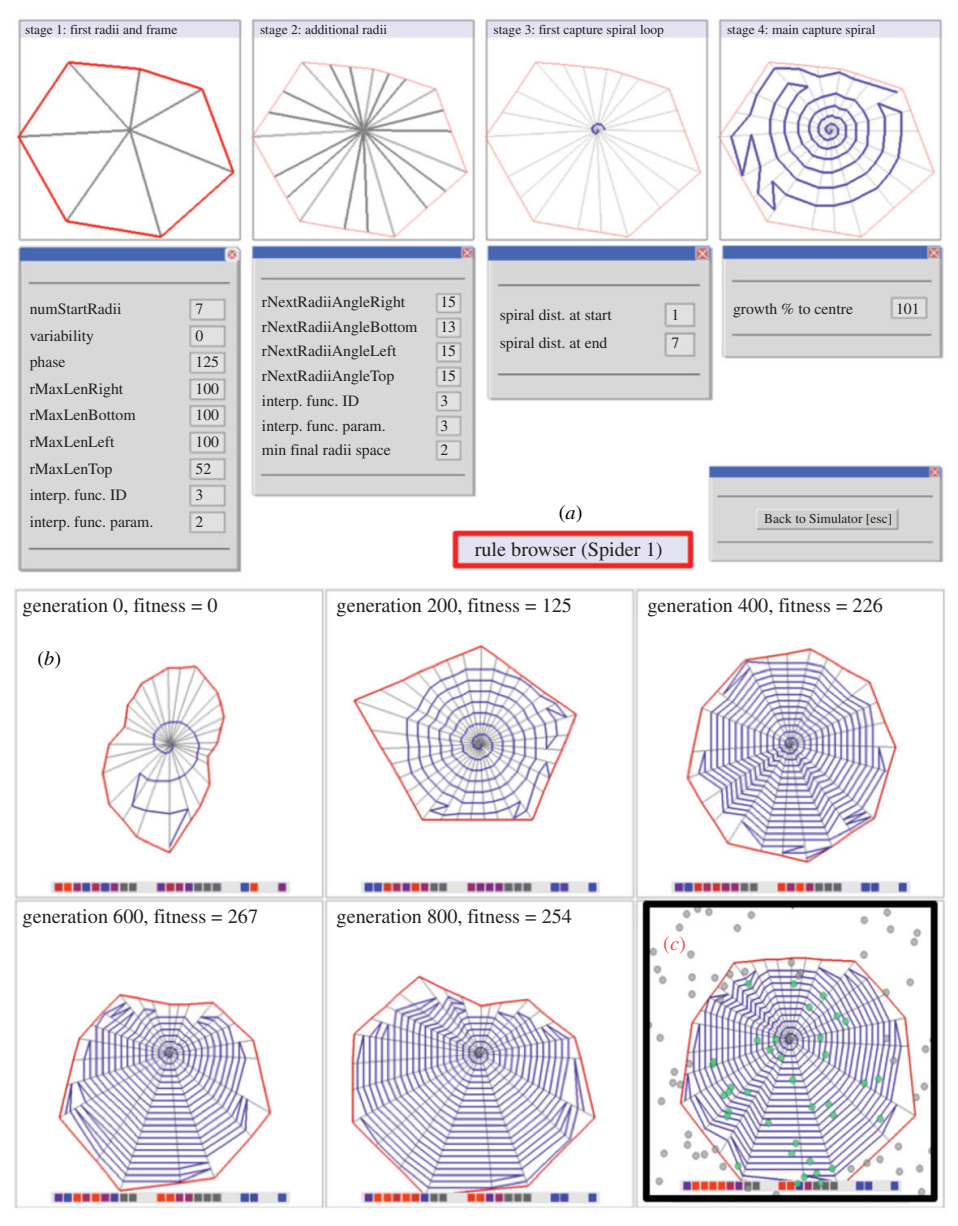
*Theseus EVO* uses simplified rules derived from all components of *Araneus* web construction. (*a*) Screen shots of the different rule sets used and sample settings. (*b*) A screen shot of the fossil record of 200 generations in the evolution of a simple web (shown in generation 1) responding to a fairly high density of small prey (inset, prey are yellow dots) with a positive bias towards prey below the hub leading to an asymmetric web. The coloured dots stripe represents the setting of the genome with each position being a gene and the colour indicating its value (with red at maximun and blue at minimum and grey that the genes were inactive and unused by this set of rules). For more on the selection process, see appendix A; electronic supplementary material, appendices SB and SD.

*Theseus AD* was surprisingly informative during the simulation and testing of a range of web-building decision rules and variables. A variant version of the cyber spider, *Theseus EVO,* enabled us to go one step further than testing details of the algorithm for spiral construction in real spiders. *Theseus EVO* allowed us to study evolutionary processes via a generic set of web-building rules extracted from observations of all stages of *Araneus* web construction starting with the frame threads and ending with the capture spiral. In this set-up, the rules were selected in sequences of simulations to allow the behaviour and thus the web to adapt in a virtual ecological niche (figure 6). Here, the cyber web carried some of the costs of a real web, specifically the energetic costs (which are different) of the silks for the radials (non-sticky) and for the capture spiral (sticky) as well as the time spent laying down (which is equal) those silks to create the web. In return, the web provides the spider with benefits embodied in our selection arena from the energy provided by the size of the prey and in some scenarios also the impact position in the web, which affects the probability of capture by the spider. The interaction of these two parameters determines the cost–benefit balance.

To allow *Theseus EVO* to respond to its environment, we employed the setting of an evolutionary (genetic) algorithm (EA or GA), i.e. a computerized optimization process modelled on the mechanisms of natural selection [35–37]. Fundamentally, an EA optimization procedure is based on the principle of encoding the parameters of a fitness function (the optimization problem) in such a way that they behave like genes in organisms, i.e. that they replicate, mutate, recombine and submit to selection. The central goal both in Nature and in an EA is for each unit (animal or bot) to accumulate resources to produce as many offspring as possible. This has to be done in competition with other units with the same goal, all aiming for more or less the same resources at more or less the same time. Comparable to any challenge, there are better or poorer solutions leading to the establishment of a new concept in the wider population or its eradication.

Natural evolution tends to be rather complex, although the underlying processes of selection are simple. The fitness of an individual, which can be defined by the number of surviving and further reproducing offspring, is determined by various factors. Among those are internal variables, such as behaviour, anatomy and physiology, as well as environmental variables such as resources (food, water, shelter), climate, predator presence, breeding opportunities, etc. In an orb spider like *Araneus*, reproductive fitness is largely determined by the number and quality of prey a spider catches during its lifetime. The more prey a female catches and eats, the larger her size and weight allowing for better web defence against potential usurpers and also, most importantly, increasing the number of eggs she can ultimately lay [38]. Thus, reproductive fitness depends on how well a web is built for catching prey. Importantly, web construction has to be not only competent but also efficient because, firstly, silk is a costly and limited resource and, secondly, web building has to be fast as it exposes the spider to the sight of predators and does not trap any prey unless it is finished and functional [39].

**Figure 7.**
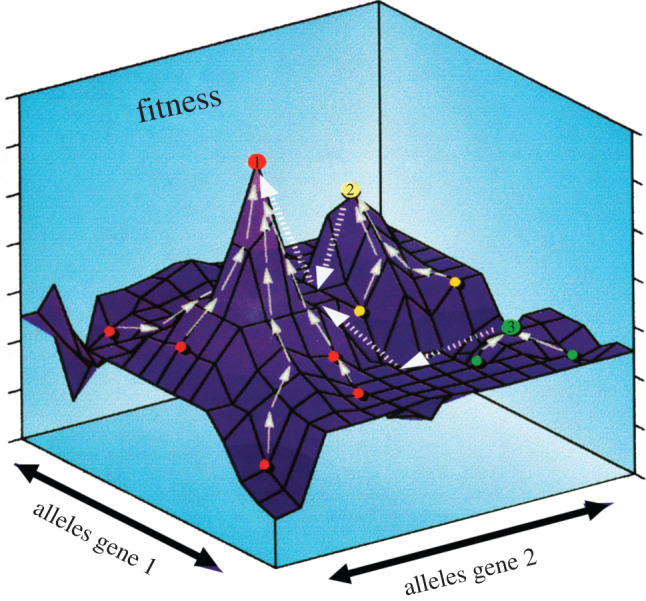
*Theseus EVO* webs responding to an evolutionary algorithm. Schematic illustration of a meta-population with three Demes (red, yellow, green) climbing independently towards their respective local peaks (1–3) in the fitness landscape (*z*-axis) and the track of the overall population finding the global peak because of inter-breeding between the Demes (web relevant alleles in *x*,*y* axes). For details, see electronic supplementary material, appendices, with SC and SD providing the outline of downloadable applications.

The evolution of a spider's web is a long and convoluted process as amply demonstrated by studies and heated discussions of orb spider phylogeny [11,12]. How much and what kind of prey gets entangled in a web and can be caught by a spider is partially determined by judicious placing of the web in the habitat and then by chance encounters with flying or jumping insects. Prey abundance, size and behaviour may vary depending on local environmental conditions and some prey might have adapted to avoid the web or escape from it. Our model allows us to simulate such environmental factors having simplified the evolutionary process such that the fitness of the virtual spider depends solely on its success of acquiring prey, given limited resources (figure 7).

## 3. Discussion

Our combination of experiments on *Araneus* informing the modelling with *Theseus* supports the general agreement that the spider's behaviour produces a web that is well adapted, perhaps even optimized, for its function as an efficient and effective trap. But the web is much more than a particle filter and missile stop. It acts also, in parallel, as an information platform for the spider by providing dedicated, built-in sensor functionality that originates from the web's action as a self-tuning morphological computing device. Enabled by the construction material, a range of silks, which in turn belong to a class of material with archetypal soft matter properties, one might even argue that this is soft robotics at its best. Selected parameters of web form and function illustrate the spider web's relevance to bio-inspiration for future technology translation of potential interest in soft robotics. As a particle filter, the web relies on the self-tuning properties and electrostatic attraction of aqueous droplets on the capture threads [40–42]. As a missile stopping net, the web re-purposes these droplets by using their action as micro-size windlass fibre-reeling mechanisms [43]. As an information platform, the web transmits displacements [42] as well as vibrations of movements within the entire structure [44] with the vibrations, filtered and tuned by the web structure, providing signals that allow the spider to remotely monitor web activity as well as web status [44,45].

Instrumental for the function of the web is its adaptability, which relies on its origin, i.e. the properties emerging from long sequences of inherited but adaptable behaviour actions, as discussed earlier. Behaviour has the benefit of being much more flexible than morphology with rapid-response sensor and actuator feedback loops aiding the controller. Focusing on a natural construction behaviour with a clear-cut cost–benefit function allowed us to discover, enable and explore a construction algorithm for an optimized net morphology that is indeed very soft, i.e. easily stretches and distorts during both manufacture and operation.

While the cost–benefit analysis of time/material expenses versus energy income (prey size/density) is a very real one for spider and robot alike, other parameters of the web engineering and architecture were not included in this study. Nevertheless, we propose that our study lays the groundwork for developing, testing and optimizing novel bioinspired behaviour algorithms for the control of engineered robots in a wide range of environments and applications. Of specific interest to the emergent field of soft robotics will be the intimate interaction of the hard-shelled arthropod animal with integrated soft sensors and the super-soft visco-elastic silk web material. Students of stigmergy will find inspiration in the functional complexity emerging from rule-of-thumb iterations. A good understanding of such complex and typically nonlinear interaction appears to be inspiring novel developments in robotics [46,47].

Thus, the analysis of spider web building provides the engineer and roboticist with a fine example for the concept of stigmergy [48] where complex, virtually intelligent structures can be created without direct interaction, communication, control or overarching planning. In computer engineering, the concept of stigmergy serves as inspiration for applications in wide-ranging areas of animal and human behaviour wherever an action configures the following action by the intermediary of the trace it leaves [49]. Examples for the phenomenon range from ant nest construction [48,50] and biofilm self-organization [51] to robot swarm behaviour [52] and satellite clusters [53] as well as the development of free open source computer programs [54].

## 4. Conclusion: reduction to practice

It will be interesting to further examine the possible applications of the combination of a real robot spider controlled by a bioinspired (i.e. soft robotic) brain and extending its phenotype into a housing using a soft-material web construction. With that in mind, we include in the electronic supplementary material links to the outcome of our research for downloading as apps and we are open to discussing and sharing code. One practical use of our study in robotics applications might be the construction and deployment of extensive net structures [55], for example, to catch space junk [56] where a new web tailored to requirement can be built on the spot using a spider-inspired robot and where silk might very well be the material of choice [57]. Another use could be the capture and analysis of airborne pollutants where the robot would take down the web daily for ingestion and analysis in analogy to a spider who recycles the web protein while also harvesting tiny insects as well as, unfortunately in some cases, pollutants [58]. A third use might be the use of temporary and energetically inexpensive scaffolds for lifting and shifting operations [46]. Indeed, this particular application could be extended to exploration and work in a denied environment where a single or swarm robot uses its web not only as a navigation and operational platform but also as an information network and energy provider—just like its inspiration, the spider, does.

## Supplementary Material

Outline of the Evolutionary Algorithm used to the cyber spider <i>Theseus EVO</i>

## Supplementary Material

<i>Theseus AD</i>, an application to study the behavior of spider orb web-building.

## Supplementary Material

<i>Theseus EVO</i>, an application to study the evolution of spider orb web-building.

## Supplementary Material

Environmental factors affecting a spider's web.

## Data accessibility

Electronic supplementary material supporting this article is available through download at http://users.ox.ac.uk/∼abrg/apps/ pointing to the outcome of our research for downloading applications with the additional comment that we are always open for joint fundamental research using our technology and also for joint software development.

## Appendix A. Effect of prey size and position in the webs of *Theseus*

The size of dots represents the size of the virtual flies. *Araneus* spiders build vertical webs and are better at catching prey in the lower (south) regions of a web partly because of gravity and partly because they already sit at the hub facing down. This behaviour can be modelled in *Theseus* and the various effects are shown in the figure where a slightly higher value is given to flies stuck to southern threads. Note that the spider responds over generations by expanding the southern regions of the web which leads to up–down asymmetries.

The coloured stripe at the bottom of each image represents the setting of the genome with each position being a gene and the colour indicating its value—with red at maximum and blue at minimum, and grey that the genes were inactive and unused by this set of rules.
