# Supplementary material for: Spider webs inspiring soft robotics
Source: J R Soc Interface. 2020 Nov 11;17(172):20200569. doi: 10.1098/rsif.2020.0569 (PMC7729045; doi:10.1098/rsif.2020.0569)
Supplement: Outline of the Evolutionary Algorithm used to the cyber spider Theseus EVO [file rsif20200569supp1.pdf]

Theseus Evo - Evolutionary Algorithm

```
initialize
    generate random population
    evaluatePopulation

evolve
    repeat until abort
        nextGeneration
        evaluatePopulation

nextGeneration
    selectPopulation (tournament selection of size 2)
        for each individual
            pick a random competitor and get replaced if he is better

    recombinePopulation
        for each individual
            with probability c_p
                pick a random partner and mix his with my genes

    mutatePopulation
        for each individual
            for each base (binary) of my genome
                with probability m_p
                    flip a base (switch 0 to 1 or 1 to 0 respectively)

evaluatePopulation
    generate prey
    for each individual
        build web
        count how much prey gets caught
        calculate and store fitness
```

Fitness calculation

Fitness = numPreyCaught * preyValue - silkCost - timePenalty

numPreyCaught	= number of prey caught in the web
preyValue	= 20
silkCost	= silkCostFactor * (capSpiral length + radii length + frame length) / 200
timePenalty	= timePenaltyFactor * (capSpiral nodes + radii nodes + frame nodes) / 20

silkCostFactor	= 10
timePenaltyFactor	= 0

Other model parameters:
Population size = 24
Number of prey = 100

Prey size	= 1 (small), 4 (mid size), 7 (large)
Attack simulation	= “Off”, “Distance from center” or “Distance from center and gravity”
Silk limit	= 0 (off) or > 0 (total silk amount / length limit)
