# Supplementary material for: Spider webs inspiring soft robotics
Source: J R Soc Interface. 2020 Nov 11;17(172):20200569. doi: 10.1098/rsif.2020.0569 (PMC7729045; doi:10.1098/rsif.2020.0569)
Supplement: Theseus AD, an application to study the behavior of spider orb web-building. [file rsif20200569supp2.pdf]

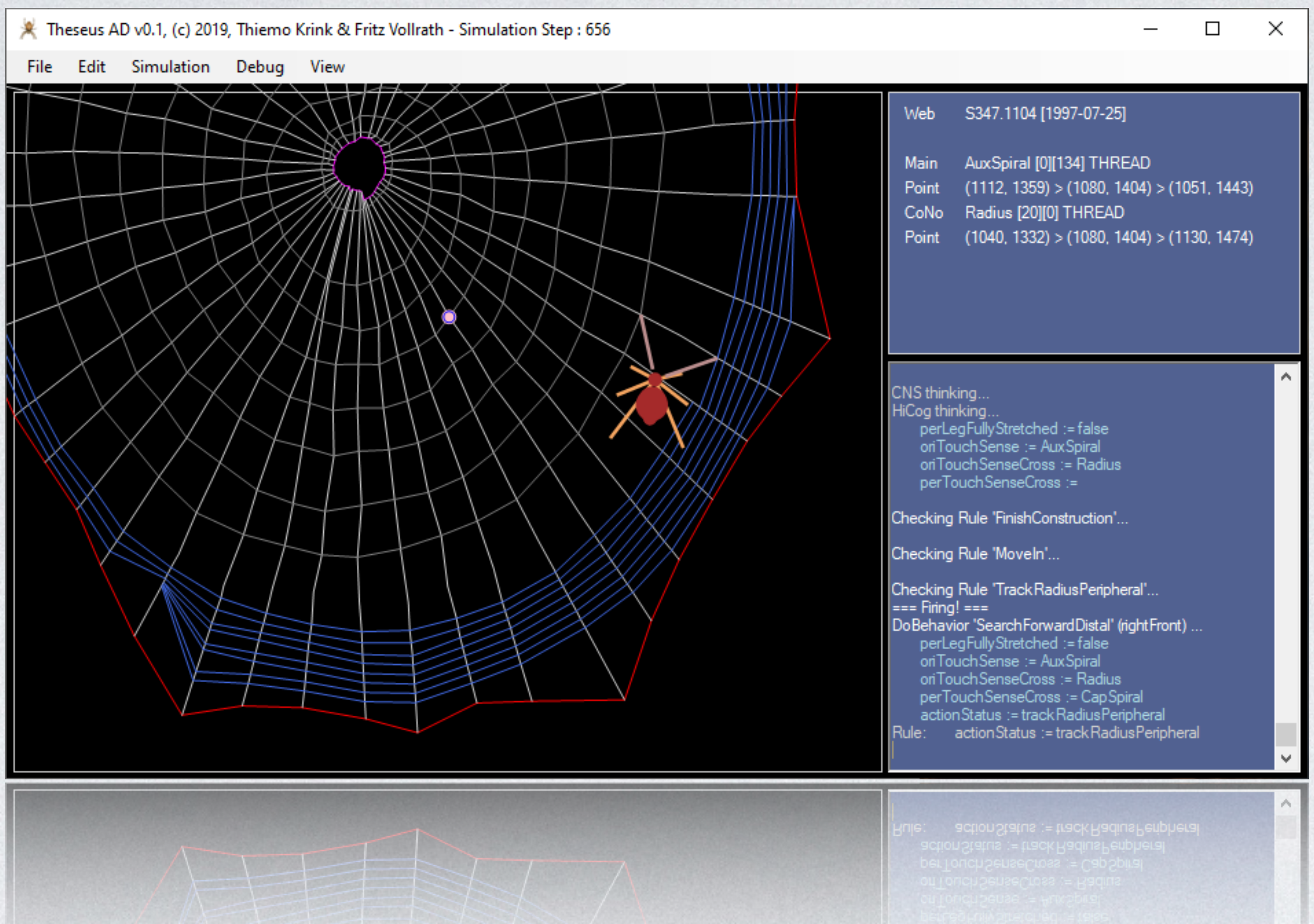

Theseus AD

Unravel Spider Behavior

by Thiemo Krink and Fritz Vollrath © 2020

Acknowledgements:

- Universities of Aarhus and Oxford
- Funding by DNRF, SRC, UKRI, EU-ERC
- TV shows in the US, UK and NL for presentation
- New Scientist and Berlingske Tidende for feature stories

Theseus AD is a hypotheses testing app to study orb web spiders (Windows PC)

App Features:

- Virtual spider robot that completes the digitized webs of real spiders
- Rule-based AI that allows to implement and test hypotheses of web-building
- Interface to load digitized webs of real spiders into the virtual world

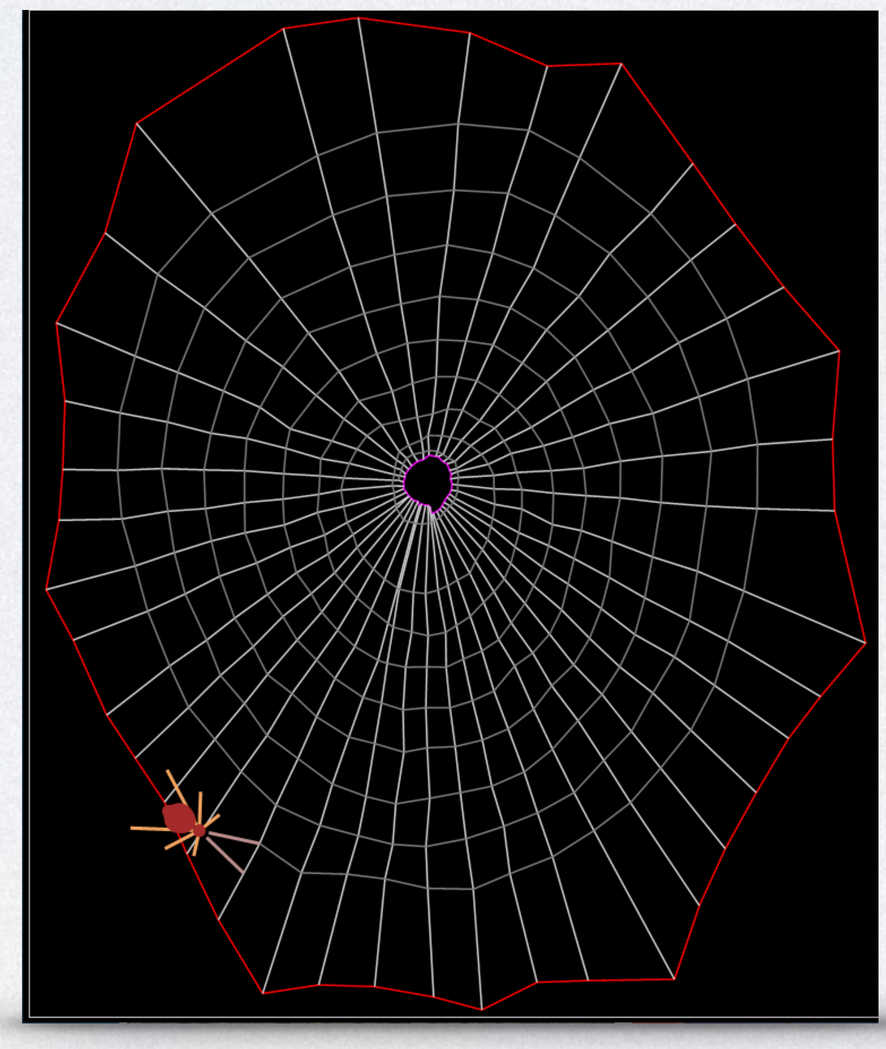

Let's go!

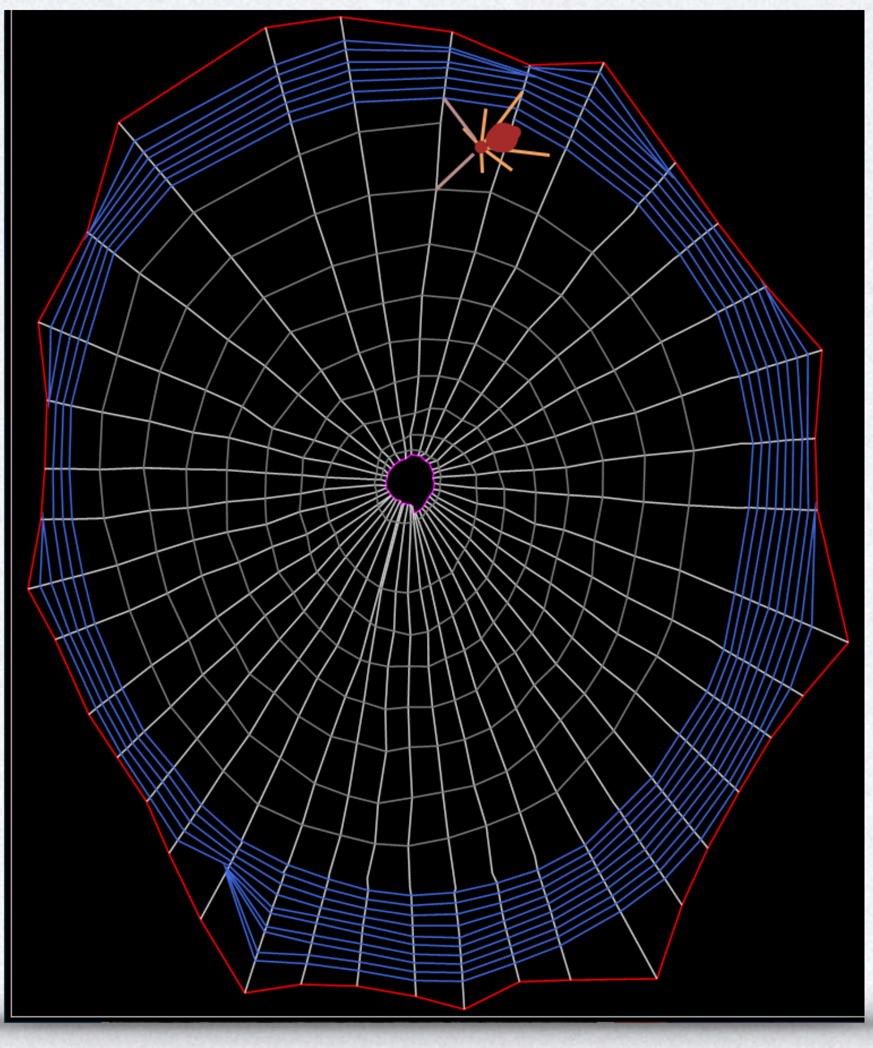

Fill-out the gaps…

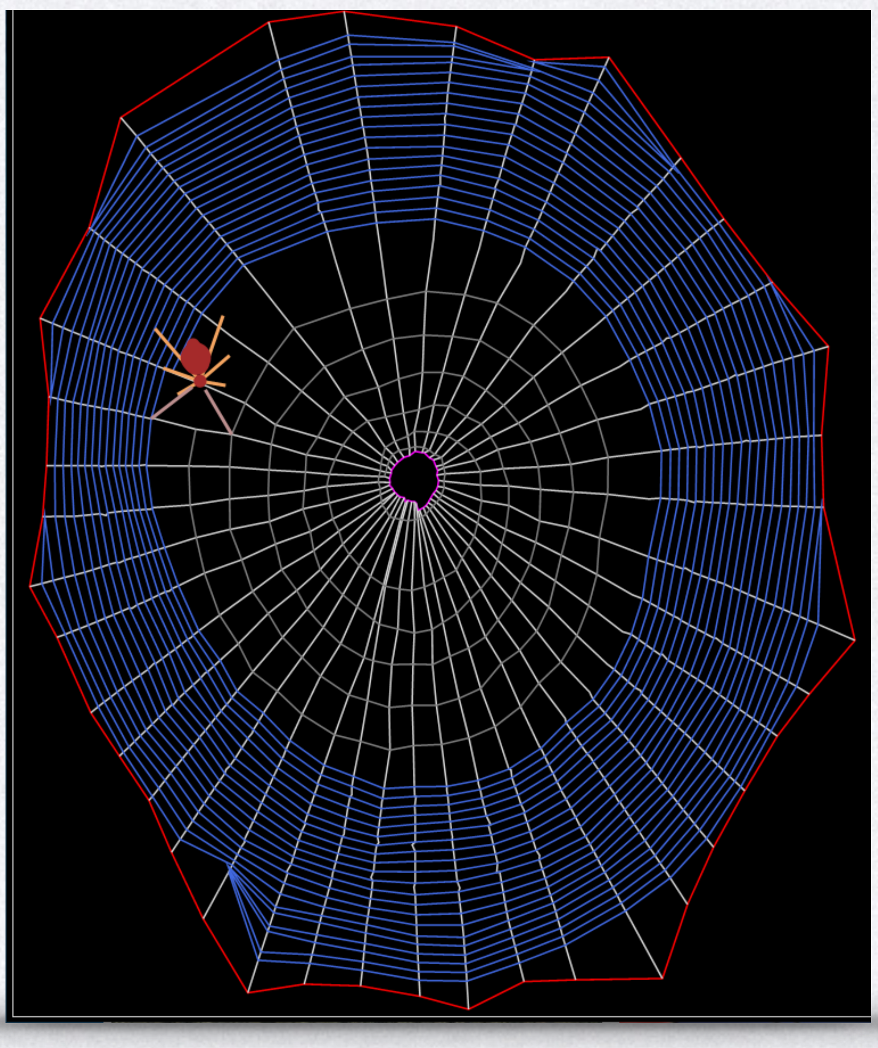

Build and take down the scaffolding…

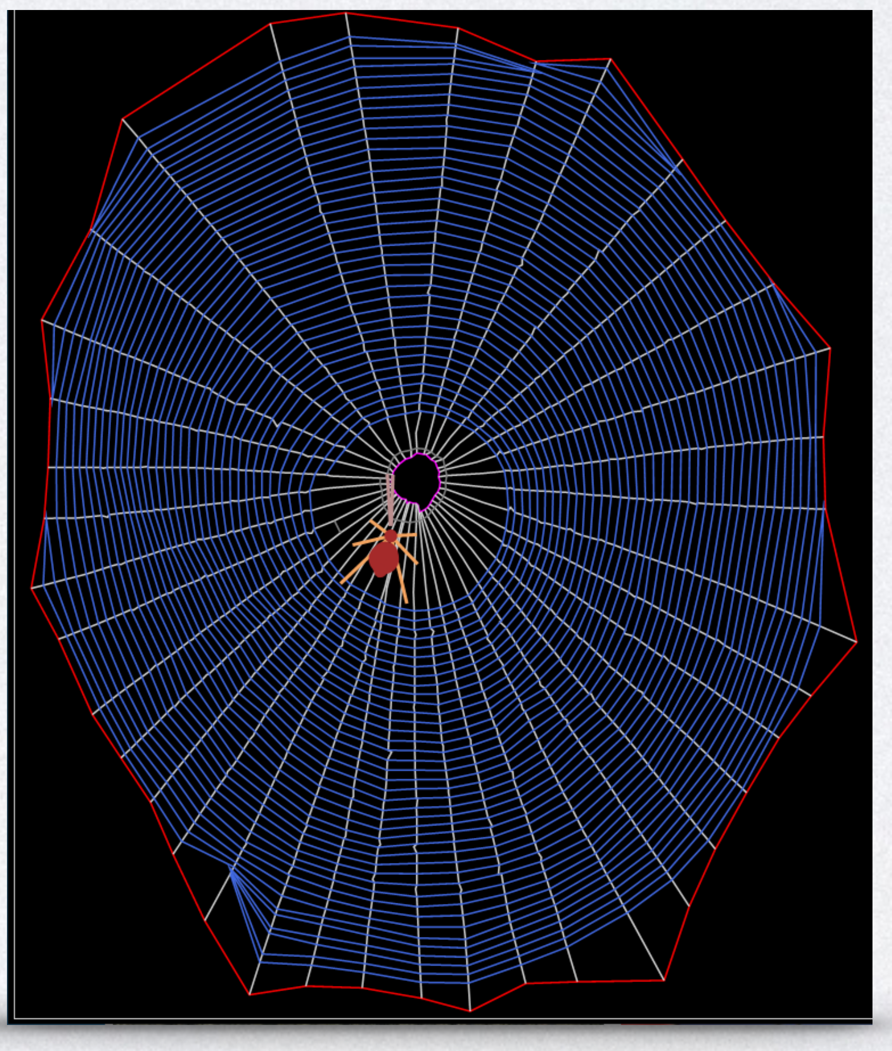

Until it's done.

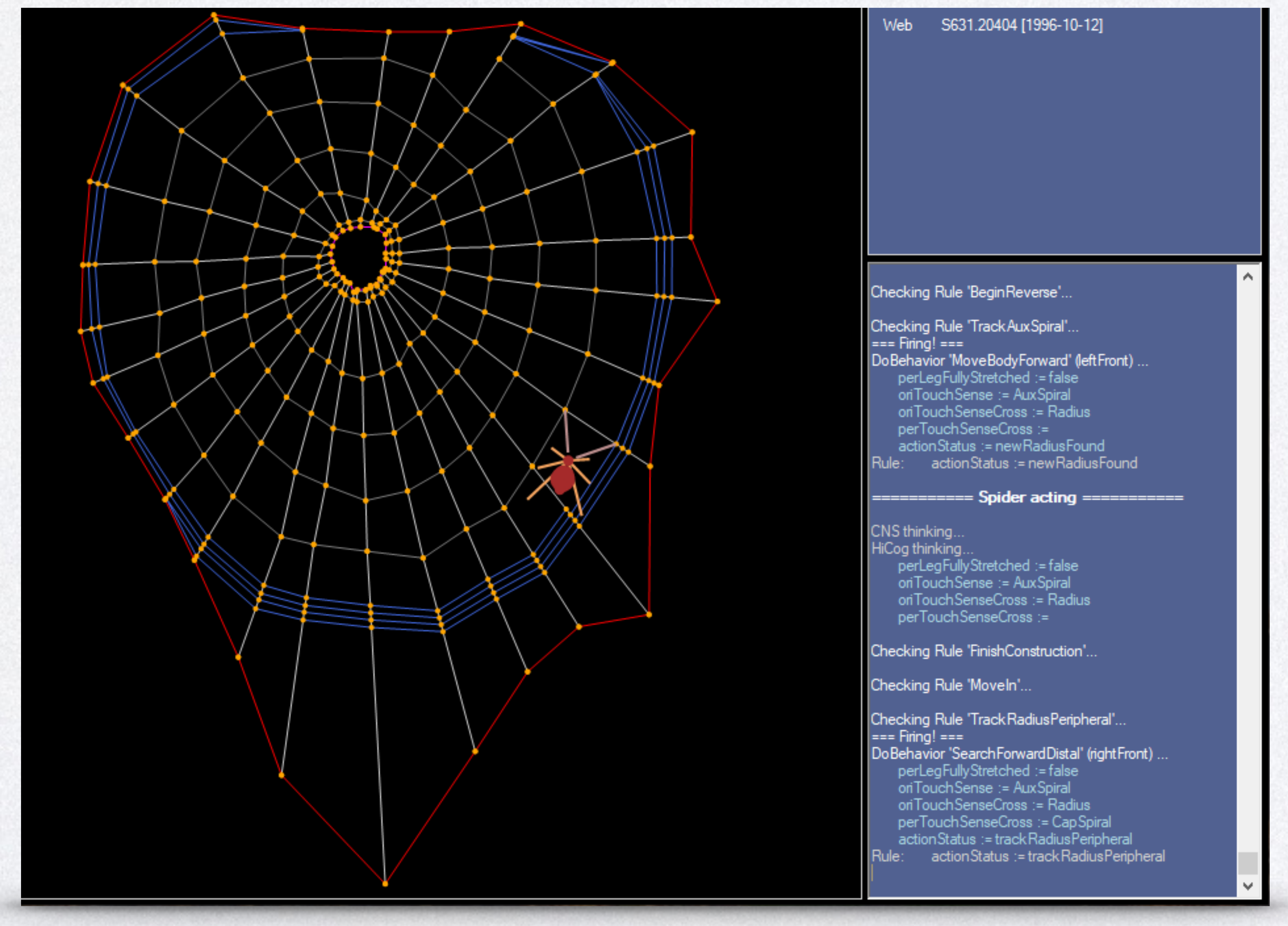

Inspect and Understand Decisions

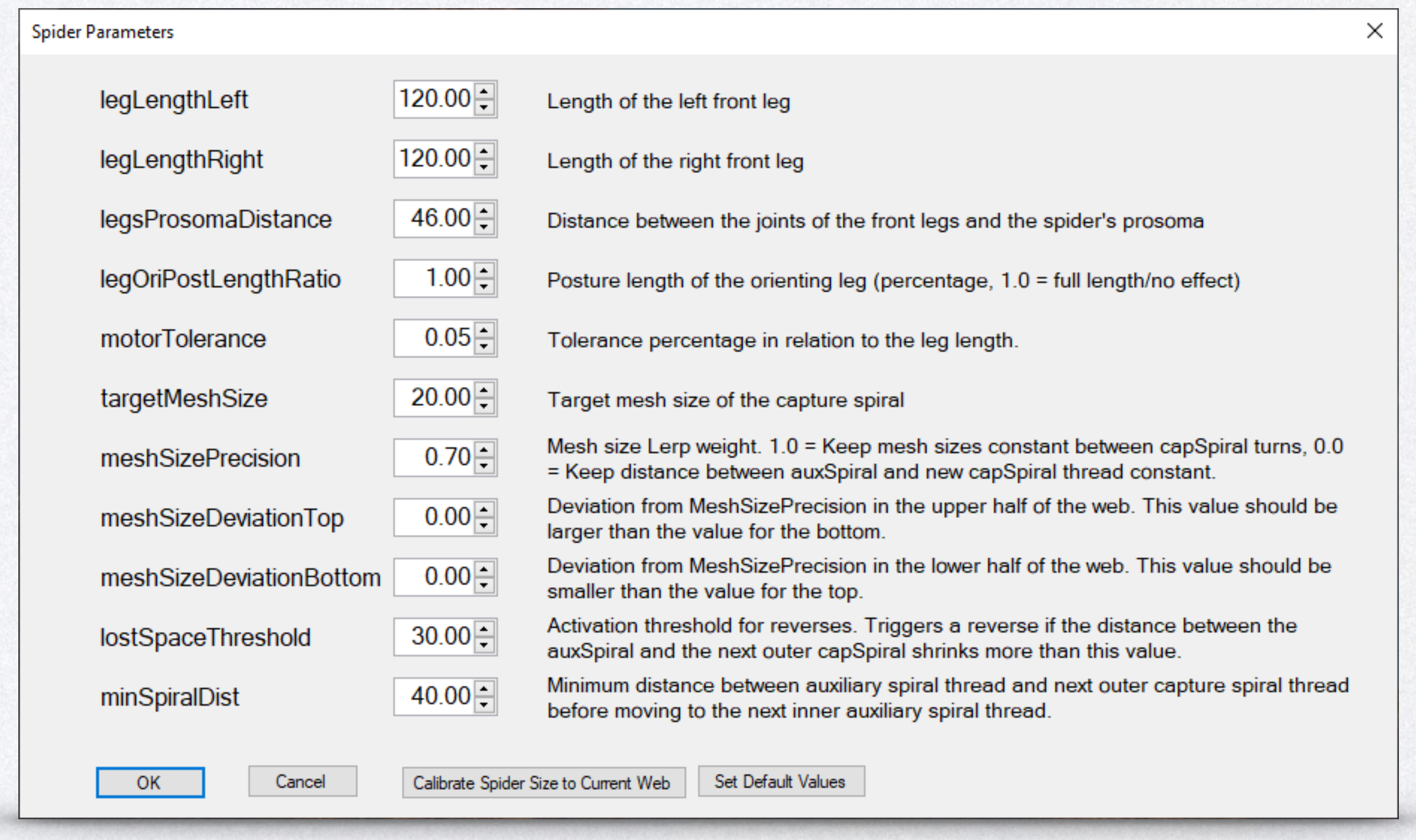

Calibrate your virtual robot
