# Supplementary material for: Spider webs inspiring soft robotics
Source: J R Soc Interface. 2020 Nov 11;17(172):20200569. doi: 10.1098/rsif.2020.0569 (PMC7729045; doi:10.1098/rsif.2020.0569)
Supplement: Theseus EVO, an application to study the evolution of spider orb web-building. [file rsif20200569supp3.pdf]

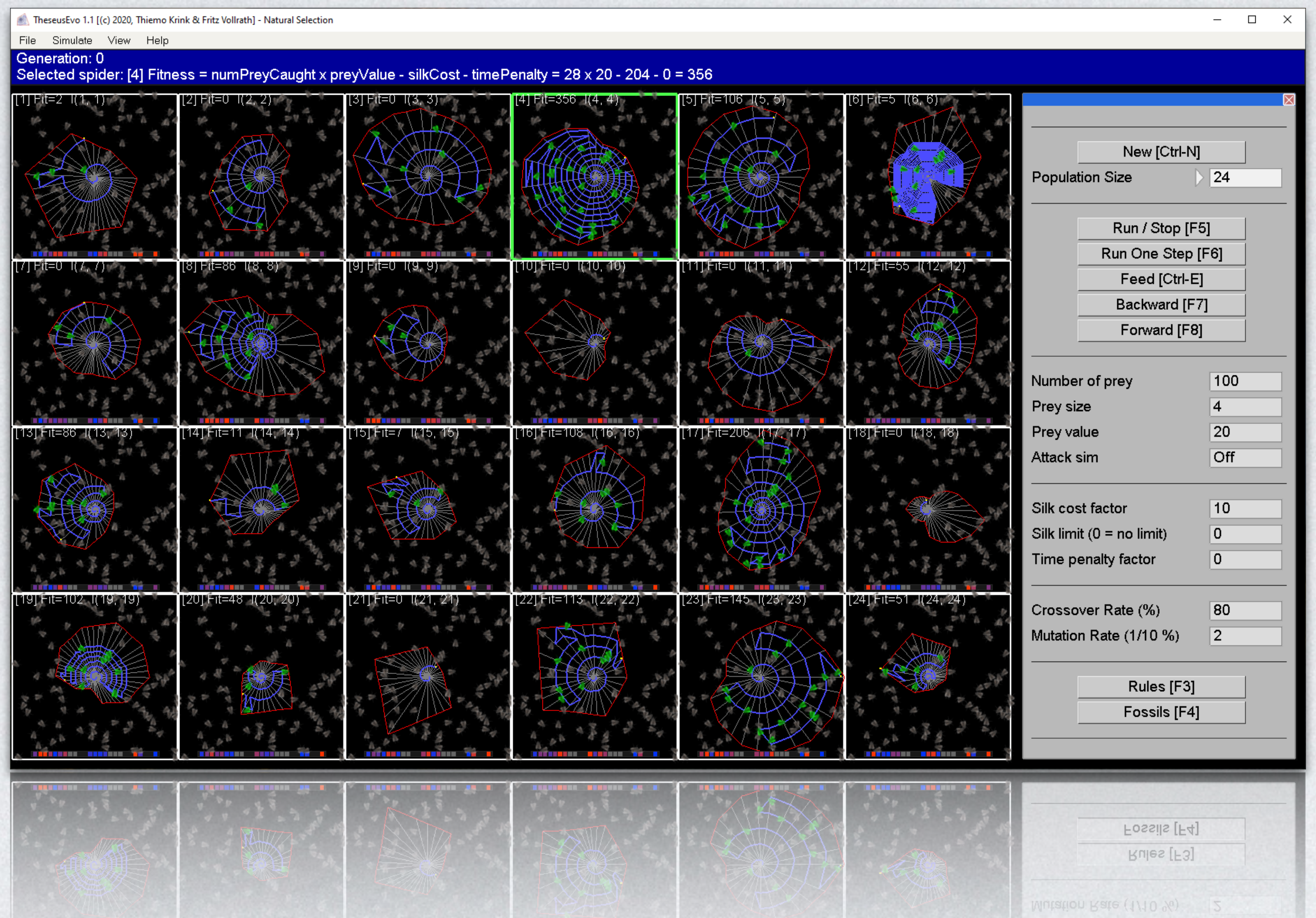

Theseus Evo

Explore Spider Evolution

by Thiemo Krink and Fritz Vollrath © 2020

Acknowledgements:
- Universities of Aarhus and Oxford
- Funding by DNRF, SRC, UKRI, EU-ERC
- Richard Dawkins see also Climbing Mount Improbable
- Presented at Royal Society Summer Exhibition 2016

Theseus Evo is a spider evolution simulator (for Windows PC)

App Features:

- Simulated Spiders with Parameterized Rules for Web Building
- Spider Evolution towards a User-Defined Niche using Evolutionary Algorithms
- Fossil Record of Spider Evolution
- Editable Genes of Web-Building Rules

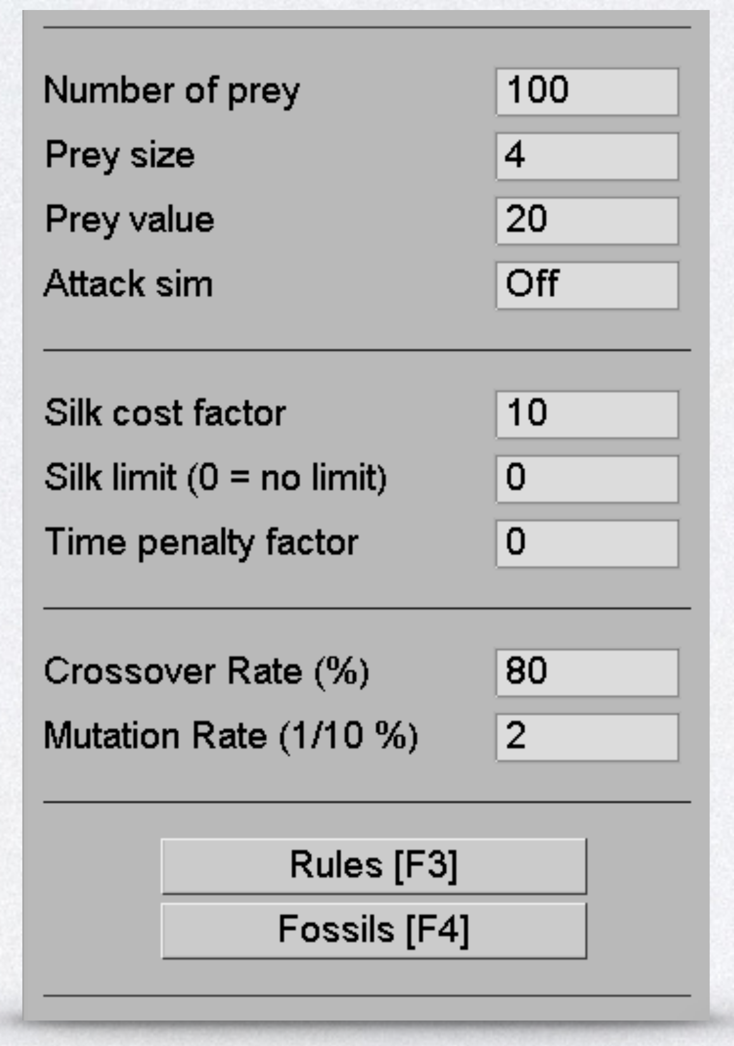

Create your niche…

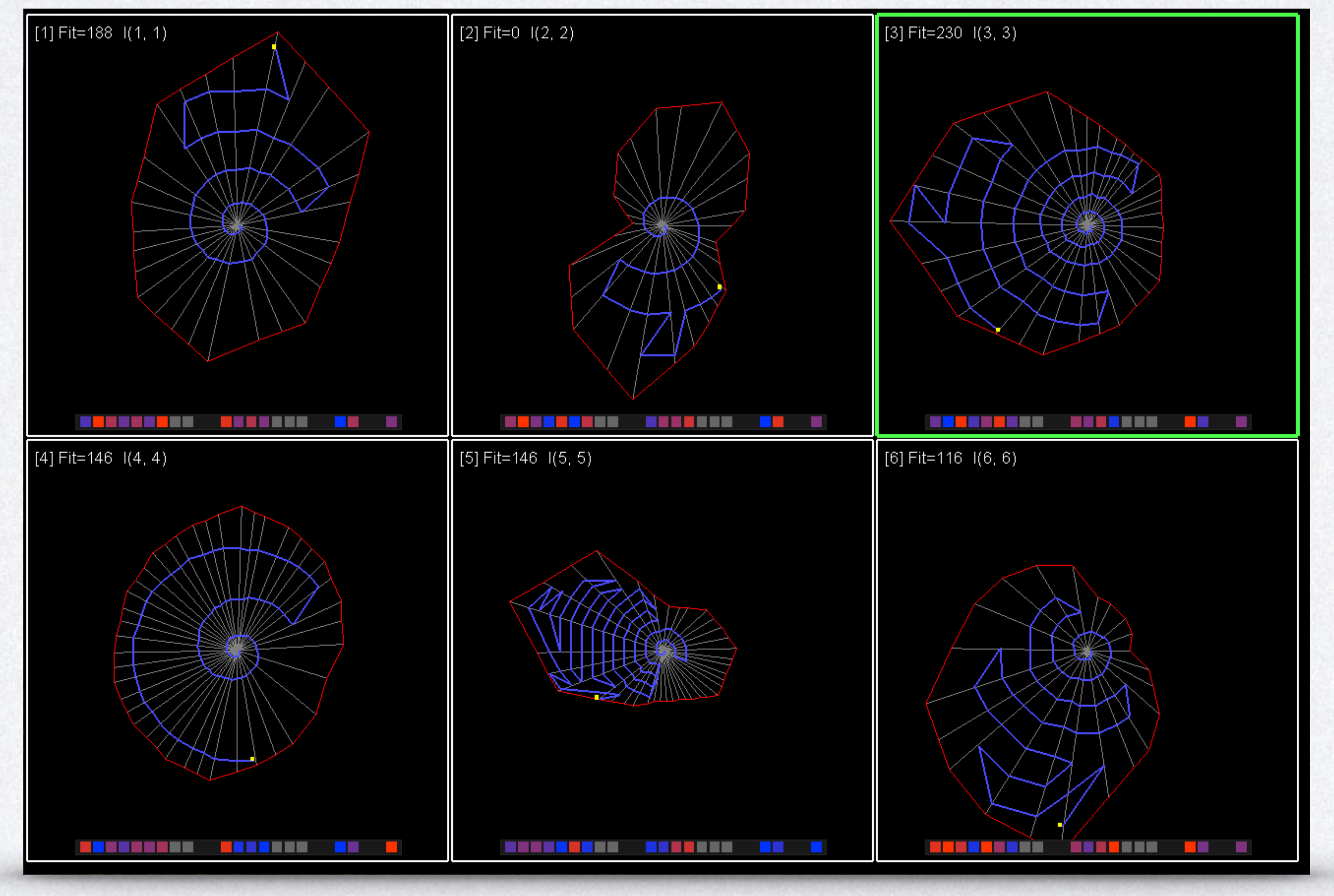

Breed…

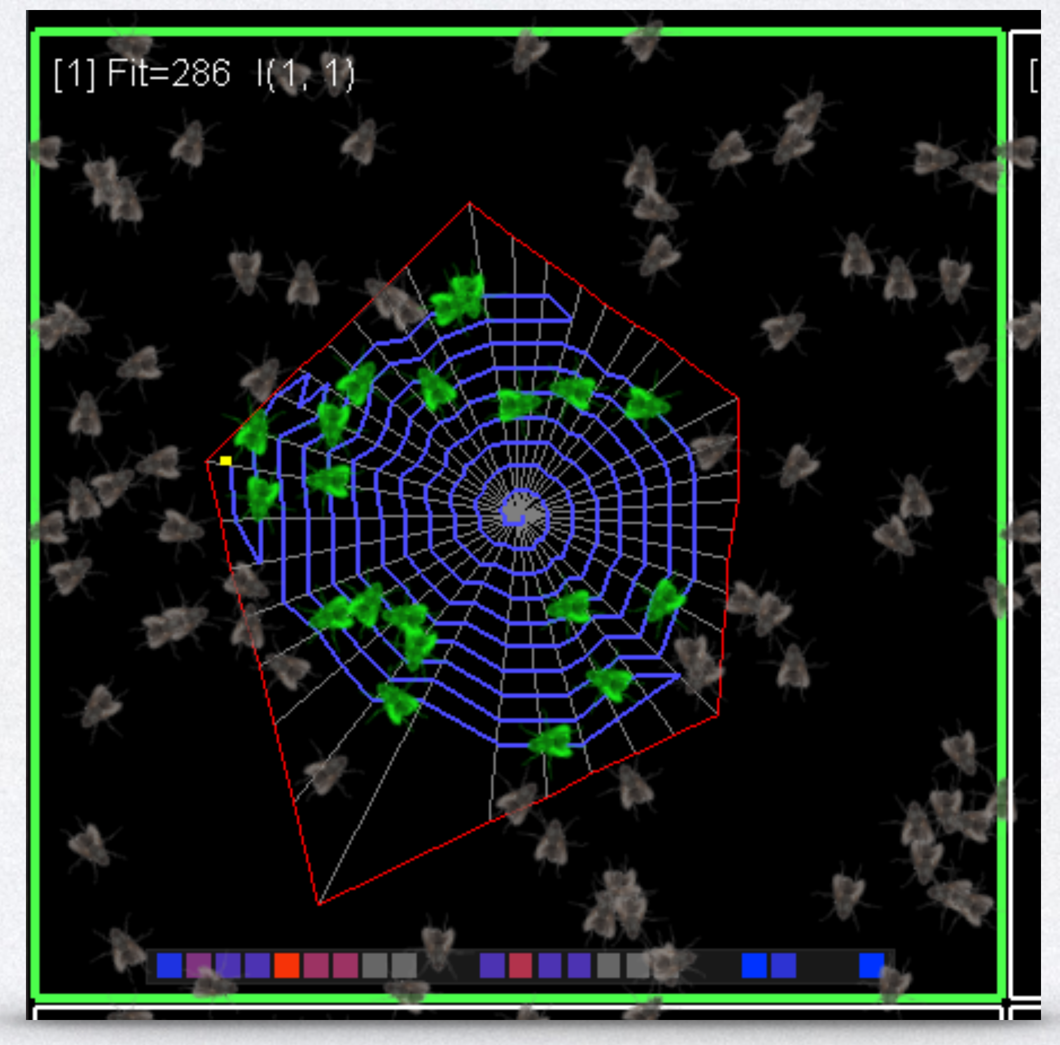

Feed…

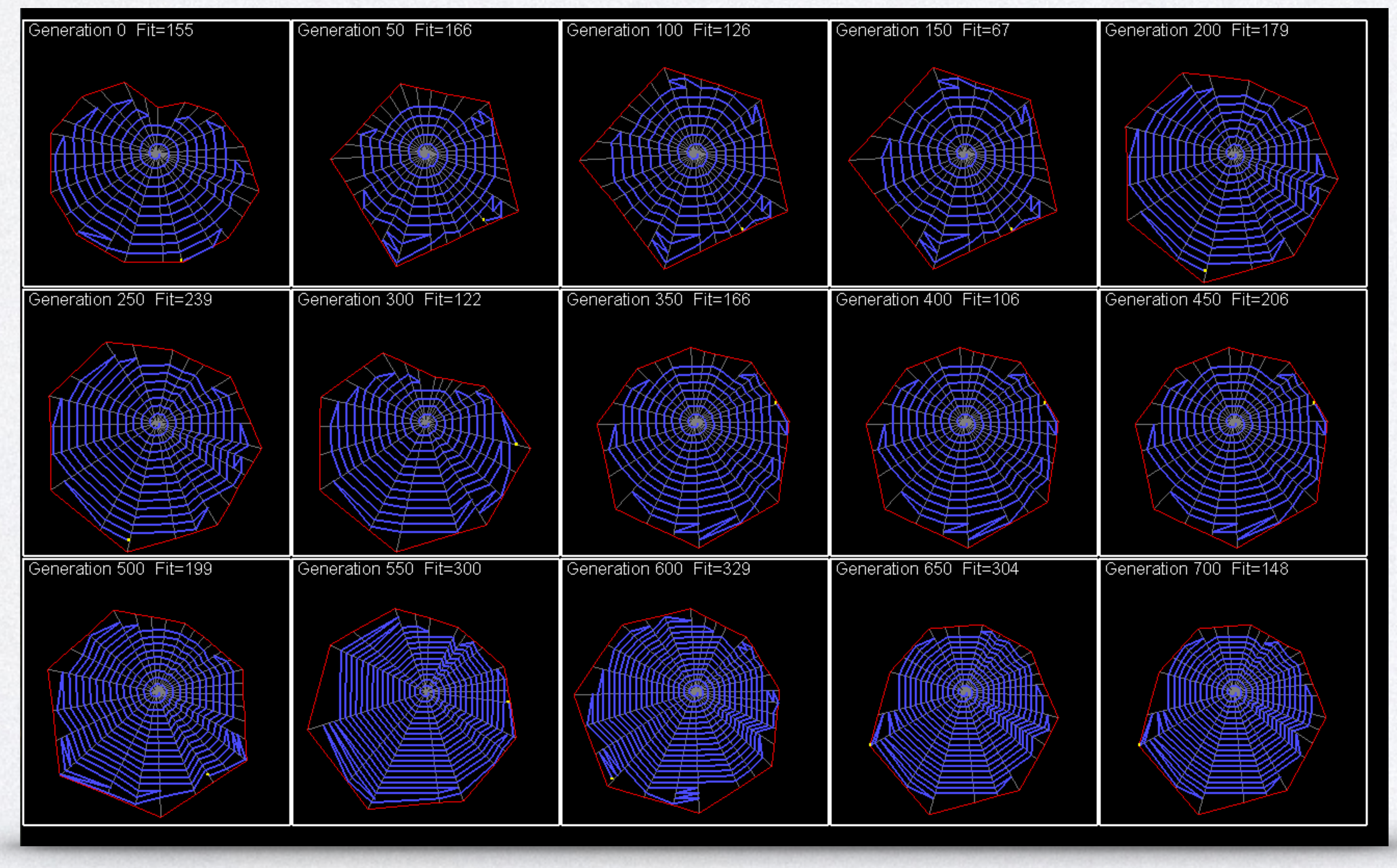

Evolve…

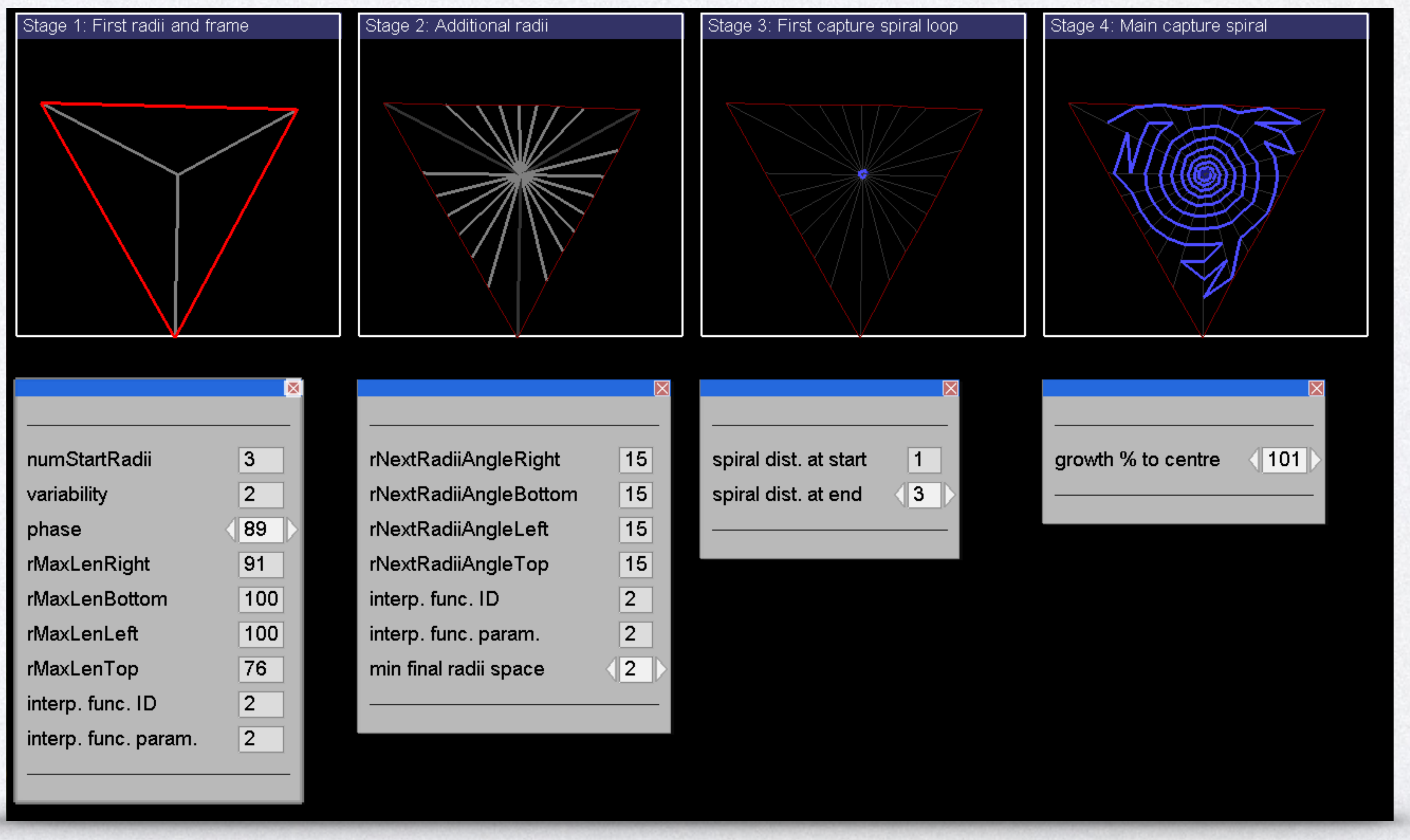

Explore The Rules!
