# Supplementary material for: Spider webs inspiring soft robotics
Source: J R Soc Interface. 2020 Nov 11;17(172):20200569. doi: 10.1098/rsif.2020.0569 (PMC7729045; doi:10.1098/rsif.2020.0569)
Supplement: Environmental factors affecting a spider's web. [file rsif20200569supp4.docx]

**Spider Webs inspiring Soft-Robotics.**

Fritz Vollrath and Thiemo Krink

**Supplementary Material:**

**SM 1** Factors affecting function and form of the orb web of *Araneus diadematus*.

The garden cross spider *Araneus diadematus* readily adapts its orb web architecture to environmental conditions with many factors affecting web construction and consequently web structure (1,2,3,4). Conditions include internal variables such as the spider’s weight, size, state of hunger and senescence as well as experience (5,6,7,8). They also include external parameters both biotic such as prey type and abiotic such as wind, humidity, temperature and building site (9, 10,11,12). And, since the same spider builds many webs during its life, typically a new web every day, one can conduct many experiments on individual web genotypes to probe their expression from gene to morphology (13,14,15). A key book (in press at the time of writing this) giving many most valuable insights will be, I am sure, Bill Eberhard’s *Spider Webs* (16).

References:

1. Tilquin A. 1942 La Toile Geometrique des Araignees. Paris: Presses Univ. France. viii + 536 pp.
2. Shear WA (ed.) 1986 Spiders: Webs, Behavior, and Evolution. Stanford University Press
3. Craig CL 2003 Spiderwebs and Silk: Tracing Evolution from Molecules to Genes to Phenotypes. OUP (ISBN: 9780195129168)
4. Herberstein M. 2011 Spider Behaviour: Flexibility And Versatility. CUP Cambridge
5. Vollrath F, Köhler T. 1996 Mechanics of silk produced by loaded spiders. Proc. Roy. Soc*.*, 263: 387-391
6. Vollrath F, Samu F. 1997 The effect of starvation on the web geometry of an orb weaving spider. Bull. Brit. Arachnol. Soc., 10: 295-298
7. Anotaux M, Toscani C, Leborgne R, Chaline N, Pasquet A. 2016 A Time till death affects spider mobility and web-building behavior during web construction in an orb-web spider Current Zoology, 62: 123–130,
8. Heiling A, Herberstein M. 1999 The role of experience in web-building spiders (*Araneidae*). Anim Cogn 2:171–177
9. Vollrath unpublished data,
10. Lin L, Edmonds D, Vollrath F. 1995 Structural engineering of a spider's web. *Nature*, 373: 146-148
11. Vollrath F, Downes M, Krakow S. 1997 Design variability in web-geometry of an orb-weaving spider. Physiol. Behav., 62: 735-743
12. Schneider J, Vollrath F. 1998 The effect of prey type on the geometry of the capture web of *Araneus diadematus*. Naturwiss. 85: 391-394
13. Krink T, Vollrath F. 2000 Optimal Area Use in Orb Webs of the Spider *Araneus diadematus*. Naturwiss., 87: 90-93.
14. Herberstein M, Tso I-H. 2011 Spider Webs: Evolution, Diverstity and Plasticity. pp 57-98 in Herberstein Spider Behaviour: Flexibility And Versatility. CUP Cambridge
15. Hesselberg T. 2014 The Mechanism behind Plasticity of Web-Building Behavior in an Orb Spider Facing Spatial Constraints. J. Arachnol. 42: 311–314.
16. Eberhard W. 2020 Spider Webs: Behaviour, Function and Evolution. University of Chicago Press.
